# Managing Epilepsy by Telemedicine in Resource-Poor Settings

**DOI:** 10.3389/fpubh.2019.00321

**Published:** 2019-11-12

**Authors:** Victor Patterson

**Affiliations:** Visiting Neurologist, Nepal Epilepsy Association, Kathmandu, Nepal

**Keywords:** epilepsy, untreated epilepsy, epilepsy treatment gap, telemedicine, teleneurology, smartphone application, APP, LMICs

## Abstract

Epilepsy is a common and treatable disease; in rich countries the expectation is that two-thirds of people will have their seizure episodes controlled on medication. In low- and middle-income countries (LMICs) however most people are not on treatment either because no doctors live near them or the logistics of affordable drug supply is absent. People with epilepsy then are prone to the bad effects of this disease—death, disfigurement from accidents and burns, and social problems due to the stigma with which the disease is associated. So this represents a failure of conventional face-to-face medicine. Might a telemedicine approach do better? The World Health Organization has suggested that non-physician health workers are empowered to diagnose and manage epilepsy; to do this they will need considerable medical support, which might be provided by telemedicine through the telephone, smartphone applications or a combination. This paper sets out what telemedicine does at present for people with epilepsy in LMICs and suggests how it might be developed in the future.

## Epilepsy in High-Income Countries

Epilepsy affects about one person in every 200 throughout the world so is one of the commonest neurological diseases. It is due to intermittent paroxysms of disordered electrical activity in the brain causing loss or alteration of consciousness and usually a convulsion (these are called epileptic seizures). Although epilepsy can be associated with other structural brain disease most people with it have only epilepsy.

Epilepsy is treatable with medication; about two thirds of people with it (PWE) have no seizures on treatment but the treatment usually needs to be continued even when seizures have stopped. In this respect it is no different from other long-term conditions like diabetes mellitus. In high-income countries doctors are plentiful and accessible and there are usually insurance schemes which cover the costs of the medication. Sadly this isn't the case in low- and middle-income countries (LMICs).

## Epilepsy in LMICs

This is where most PWE in the world live−40 million of the total of about 50 million^1^. Here many PWE are not on any treatment for their disease and this gap in treatment ranges from about 30% to over 90% (1–3). The reasons for this are shown in Table 1.

**Table 1:** Main causes of the epilepsy treatment gap^3^.

Belief that epilepsy is not a medical condition
No access to doctors
Unable to access medicines consistently
Unable to afford medicines

Lack of access to doctors is an important cause (2, 3). Most patients in many LMICs live rurally whereas the vast majority of doctors live in towns and cities. Even then epilepsy is regarded as complicated by most doctors and many doctors will not treat it so PWE will have to go even farther from home for treatment.

For example, in the district of Myagdi in Nepal the principal town, Beni, is 2 days travel from the northern part of the district. But there may be no doctors to treat epilepsy there so that means another half-day travel to Pokhara, the nearest large town. If a neurologist is to be consulted then there will be need for another day's travel to Kathmandu. So this is a return journey of 1 week simply for a consultation. Most people are subsistence farmers and can afford neither the travel costs nor the time away from their land. Instead they will consult the traditional healer who at least will live nearby.

## Effects of Epilepsy

The effects of epilepsy on individuals and families can be considerable. First epilepsy is a killer disease. PWE are much more likely to die prematurely than unaffected people and death is more likely if people are untreated (4, 5). Extrapolating these figures probably about 250 000 people die each year of epilepsy in the world which is a fairly staggering number. It attracts almost no attention even though it is a much greater number than the death toll from the 2014-16 Ebola outbreak in West Africa which attracted global headlines. This is because epilepsy deaths are diffused both in time and throughout the world. Unlike Ebola they don't occur in clusters and they are not much talked about. The commonest causes of death in which epilepsy is a factor are shown in Table 2.

**Table 2:** Causes of death in epilepsy.

Falls
Drowning
Status epilepticus
Sudden Unexplained Death in Epilepsy (SUDEP)
Suicide

As well as deaths there are two other important consequences of having epilepsy, injuries during a seizure, and stigma. Burns are the commonest serious injury and can have devastating effects especially where burns units are few. Stigma is the reaction which other community members have toward someone with epilepsy which is predicated by their beliefs about the condition. Often this results in PWE and their families being shunned or excluded from school often because the condition is thought to be contagious. This is well-shown in the docu-drama “Juneli” produced by the Nepal Epilepsy Association^2^.

## Time for a Rethink—a Public Health Approach

Although epilepsy in general is regarded as a neurological problem it might be more useful to regard *epilepsy in LMICs* as a public health problem, and involve not just the specialists who treat individual cases, be they neurologists, psychiatrists, pediatricians or physicians, but public health doctors as well. This approach would deal with epilepsy in the same way as malaria and HIV/AIDS.

To do this the individual steps for both community and individual management need to be set out, and then ways of dealing with each of them determined. Once these steps are identified then solutions to them can be devised, tested, and funded. This is essentially the approach that has been used in HIV/AIDS. One such scheme, under the acronym AIDARP, is shown in Table 3.

**Table 3:** AIDARP—a public health approach to epilepsy.

Awareness
Identification
Diagnosis
AEDS and education
Review
Prevention

*AED, anti-epileptic drug*.

Diagnosis, treatment and review are conventionally medical issues and determining how they should be done in the absence of doctors needs an innovative solution. This will have an immediate effect on the burden of epilepsy in a community unlike prevention of obvious causes of epilepsy—birth injury, brain injuries, and infections such as neurocysticercosis—which may take many years to have an effect.

## Empowering Non-Physician Health Workers

This has been put forward as a solution by the World Health Organization (WHO) in a series of publications culminating in a declaration by the World Health Assembly in 2015^3^. They said “*…by training non-specialist health care providers in order to provide them with basic knowledge for the management of epilepsy so that epilepsy can be diagnosed, treated, and followed up as much as possible in primary health care settings,…*”

There are about 10 times more non-physician health workers (NPHWs) than doctors and they live much nearer PWE than doctors so this seems a good idea. But it is a very disruptive approach and one at odds with guidelines in richer countries where even specialists in fields other than epilepsy are discouraged from diagnosing and managing PWE. For example in the UK the influential Scottish Intercollegiate Guidelines Network (SIGN) guideline on epilepsy states that “*The diagnosis of epilepsy should be made by an epilepsy specialist*”^4^. This is completely impractical in LMICs if any progress is to be made to closing the treatment gap.

If NPHWs are going to take on this role they need tools which will makes their diagnosis and management as robust as possible. But first it is important to outline the steps required to diagnose epilepsy.

## Steps in Diagnosis and Management of Epilepsy

A number of questions need to be answered when a doctor encounters a patient with possible epilepsy in order to guide management. Those which I ask are shown in Table 4.

**Table 4:** Questions to be answered in the diagnosis and treatment of epilepsy.

Is the episode epileptic or not?
If so, is it provoked or symptomatic?
What seizure types are present?
What is the epilepsy type?
What investigations have been done?
What treatment is being taken?
What is the best treatment?

Of these, the first question about episode diagnose is much the most important. The distinction between epileptic seizures and the conditions which may mimic them is made entirely from the person's story and a description from an eye-witness of the episodes. There is no investigation here which helps and the diagnosis of episodes, even in the best hands, has a definite error rate of up to 18% (6).

This diagnosis is made with a series of questions about what happens before, during and after the episode. Experienced doctors use a Bayesian approach to diagnosis (although usually not consciously)—they start off with the likelihood of someone having epilepsy and then ask a series of questions which increase or decrease that likelihood. Some of these questions are likely to be more useful than others.

Determining whether episodes are epileptic or not usually takes the most time in the history; the other questions are more straightforwardly answered but again by history from the PWE and an eye-witness.

Videoclips of events can be recorded at home by patients' families and taken to a specialist for viewing, particularly in high-income countries where most patients have a smartphone. In my practice in LMICs this does not happen often, usually because patients' phones do not have the facility to capture video.

## A Smartphone Application for Episode Diagnosis

LMICs may be poor in many aspects of everyday life but they are generally not poor in access to mobile phone networks, often with data facilities. Most families possess a regular mobile phone and smartphone use is increasing rapidly. Smartphone applications (apps) therefore might be potentially useful in epilepsy care. We have developed an app for NPHWs to use to answer the question of whether an episode is epileptic or not^5^. The algorithm underlying this is based on a study from Nepal which analyzed the responses to 50 routinely-asked questions in a consecutive cohort of 67 patients presenting to an epilepsy clinic, some with epilepsy and some without it (7). It was possible for each question to calculate a Likelihood Ratio (8) of a positive answer indicating epilepsy and therefore to find the questions with the most discriminating likelihood ratios for or against epilepsy. These could then be applied sequentially to the pretest odds of epilepsy in a naïve-Bayesian way to end up with a post-test probability score of the person having an epileptic episode.

This algorithm was then converted into an app—Epilepsy Diagnosis Aid—by a software company (NetProphets Cyberworks Pvt., Noida, India). This presentation had considerable advantages over other presentation methods such as paper, an electronic calculator or a web-platform in that the records could be stored and viewed on an existing personal device which could be used offline, uploaded to an internet server when a connection was available, and downloaded for batch analysis at a later stage.

This app was then validated by NPHWs and inexperienced doctors in 132 patients with the results compared to the gold standard of a face-to-face consultation by an epilepsy specialist (9). Sensitivity was 88% and specificity 100%. The app was shown to be easily-used by 15 computer-naïve village health workers (10). In a further study these health workers used the app to diagnose episodes in 96 patients, both established and newly-presenting; their diagnostic accuracy compared to an epilepsy specialist was 92% compared to 93% obtained by non-specialist doctors (11). Thus, NPHWs can be trained to answer the first question in Table 4 using this app.

Compared to established paper-based ways of episode diagnosis this app results in far fewer misdiagnoses-−8% as opposed to 25% in the study of Anand (12) which used a pragmatically-derived algorithm. Comparative data from the WHO mhGap algorithm^6^ (also available as an app) have not been published. At present the basic version of the Epilepsy Diagnosis Aid app is available free from the Google Playstore and Apple app stores.

A similar tool for children has been described and validated (13, 14). This uses a pragmatic rather than a Bayesian approach but has not been presented as an app.

## A Combined Telemedicine Approach in Nepal

But this app on its own does not empower NPHWs to diagnose and manage epilepsy as envisaged by the WHO. It needs something added. In a study from Myagdi, the rural district in Nepal referred to earlier we have done this (15). Here we trained some local villagers without any health background in epilepsy and in using the app. We provided them with some educational materials about epilepsy and sent them back to their villages to educate their communities about epilepsy and offer treatment. When these epilepsy field workers (EFWs -they were not even NPHWs) identified someone with possible epilepsy they were able to use the app and derive a probability score. They then telephoned an epilepsy specialist in Kathmandu who was able to talk to the patient, with the EFW present, and prescribe treatment which the EFW then arranged. The crux of this method is that knowing the app score, and having confidence in it, reduced substantially the time required for history taking and diagnosis by the specialist.

This combined telemedicine approach was judged for the dimensions of quality as defined by the US Institute of Medicine (16)—safety, effectiveness, efficiency, equity, patient-centredness, and timeliness. For safety, PWE were assessed where possible face-to-face by a different epilepsy specialist to look for misdiagnosis and none was found. There was no excess mortality and reported AED side-effects were only 5% (15). Most people had significant reductions in their seizures and most patients were highly satisfied with the service which did not involve them in any significant travel. The epilepsy treatment gap was reduced from 43 to 9%, some patients opting not to take AEDs. It provided much better use of the epilepsy specialist's time.

The advantages of this approach is that the EFWs can contribute to the other public health aspects of epilepsy referred to earlier—awareness, identification, and prevention.

## Review by Telephone

Of all telemedicine techniques the telephone is perhaps the most potent especially with the advent of the mobile phone which make it both ubiquitous and portable. Its commonest use in epilepsy in both high- and low-income countries is to obtain an eye-witness account of an episode where the patient is in the clinic but the eye-witness is somewhere else. There is little published on this probably because it is so obviously beneficial. The telephone is also used extensively by epilepsy specialists in high-income countries, both nurses and doctors.

The conventional method for reviewing PWE in both parts of the world is to have them come to see the specialist at a clinic, the location of this being at the specialist's convenience rather than that of the PWE. In a recent double-blind study from a large specialist epilepsy clinic in India (17), this approach was compared with telephone review in PWE whose epilepsy was judged stable. The authors found no difference in seizure control or adherence between the two groups but found that the PWE in the telephone group had considerably lower personal costs and were less likely to default from follow-up than the conventionally-managed group.

## Email and Webservers

It is possible to exchange information between patients and doctors using email or text messaging and this is a widespread and informal method. But it is not likely to be generally applicable either because many PWE don't feel comfortable with email or their doctors don't want to share their email address.

A more beneficial method, however, is email communication between doctors or NPHWs in LMICs with specialists elsewhere. Again, this is often done informally but there are more formal systems for doing this such as the system run by Swinfen Telemedicine (18),^7^. This uses a webserver rather than email both for reasons of security and ease of record-keeping. This system, and the one run by the charity *Médecins Sans Frontières* (19), have treated PWE. These systems allow epilepsy specialists in high-income countries to contribute to the care of PWE in LMICs without leaving their offices.

## EEG

The electroencephalogram (EEG) is a system whereby the brain's electrical activity is recorded through the skull. In between episodes this is surprisingly insensitive at diagnosing epilepsy. In a study from Bhutan the sensitivity of standard EEG was only 25% and that of a smartphone-based EEG system only 17% (20). The proper use of EEG is in the evaluation of those PWE whose epilepsy is not responding to AEDs and in whom surgery is being considered. This is not the most pressing problem for epilepsy management in resource-poor countries where up to 90% of PWE are not on any treatment. Another issue with EEGs is the reporting of the record which requires both experience and expertise, qualities not always available in resource-poor countries.

EEGs are now produced in digital format which generates a file which can be uploaded to a server over an internet connection and be reported remotely by an expert. This system has been used in the UK where there is a shortage of doctors qualified to report EEGs (21). The lead author has founded a charity to deliver this service throughout the resource-poor world^8^.

## SMS Messaging

Text messaging using short messaging service (SMS) on mobile phones has been used as a way of continuing with epilepsy education in epilepsy patients under review (22). The authors of this study from Malaysia found that knowledge of epilepsy, medication adherence, and review attendance were all better in the group receiving SMS messages compared with a control group, which received conventional written information only.

## Videoconferencing

There is a common delusion in many medical circles that telemedicine equals videoconferencing. The problem with holding this view is that all the modalities mentioned above are disregarded and their potential ignored. Videoconferencing is included here because there is essentially no published work on its use in epilepsy in LMICs simply because organizing videoconferencing at the best of times is a complicated business requiring two doctors and a patient to be in the same place at the same time and the communication between them in terms of internet bandwidth to be high-quality and consistent. The former is difficult to arrange, the latter usually impossible. This is why it doesn't happen very often. In any event, for epilepsy, where the diagnosis is obtained by the history (compared to stroke where it is obtained by examination), a video adds very little.

## Future

It is increasingly clear that, on its own, conventional face-to-face medicine delivered by doctors is going to do little for epilepsy care in poorer countries so alternative ways of practice must be found. The use of the plural is important: there is no single way which will improve care everywhere and each local circumstance, in terms of location and of available human input, will require a slightly different approach. But telemedicine, with apps, webservers and the telephone, has the wherewithal to provide this necessary variety of approach; and its full potential is yet to be reached.

## Author Contributions

The author confirms being the sole contributor of this work and has approved it for publication.

### Conflict of Interest

The author holds the intellectual property rights of an epilepsy diagnosis app.

## Footnotes

^1^Available from: https://www.who.int/mental_health/neurology/epilepsy/en/

^2^Available from: https://www.youtube.com/watch?v=oLa0rlJGAck

^3^Available from: https://apps.who.int/iris/bitstream/handle/10665/251923/B136_R8-en.pdf?sequence=1&isAllowed=y

^4^Available from: https://www.sign.ac.uk/assets/sign143_2018.pdf

^5^Available from: www.epilepsyapp.org

^6^Available from: https://www.paho.org/mhgap/en/Epilepsy_flowchart.html?reload

^7^Available at: http://www.swinfencharitabletrust.org/

^8^Available at: https://www.teleeeg.org/
